# Traumatic brain injury among American indians/alaska natives in the united states: a comprehensive systematic review of the literature

**DOI:** 10.1007/s10143-025-03817-2

**Published:** 2025-10-01

**Authors:** Laurel A. Seltzer, Mohammed A. Fouda, Susan C. Pannullo

**Affiliations:** 1 Department of Neurological Surgery, School of Medicine, Tulane University, New Orleans, LA USA; 2 Meinig School of Biomedical Engineering, Cornell University, Ithaca, NY USA; 3 Department of Neurological Surgery, New York-Presbyterian Hospital, Weill Cornell Medicine, New York, NY USA

**Keywords:** Traumatic brain injury, TBI, Head injury, Alaska, American indian, Alaska native

## Abstract

Traumatic brain injury (TBI) represents a significant public health burden, particularly among American Indian and Alaska Native (AI/AN) populations in the United States, and particularly in the State of Alaska. This systematic review aims to evaluate the incidence, risk factors, outcomes, and healthcare disparities associated with TBI in this population and to identify strategies for prevention and improved management. Following PRISMA guidelines, we conducted a systematic review of Medline/PubMed, Google Scholar, Web of Science, Scopus, and Embase through May 2025. Inclusion criteria targeted studies involving AI/AN populations in the United States that reported on epidemiology, causes of injury, hospitalization, and mortality rates, as well as healthcare access. Data were extracted and synthesized qualitatively as we summarized, compared, and interpreted findings narratively based on common patterns and themes. The NIH Quality Assessment Tool was used to evaluate the quality of the included studies. Fourteen studies met the inclusion criteria. AI/ANs exhibited significantly higher TBI-related emergency visits (1,026 per 100,000), hospitalizations (186.7 per 100,000), and mortality rates (up to 65.9 per 100,000) compared to other racial groups. Leading causes included motor vehicle accidents, falls, and self-harm. Geographic isolation, limited neurosurgical infrastructure, and mistrust towards non-Native healthcare providers exacerbated disparities. Interventions like telemedicine, community health training, and culturally sensitive healthcare were identified as potential solutions. AI/AN populations, particularly those residing in the state of Alaska, face significantly higher incidence and worse outcomes from TBI due to systemic healthcare barriers and regional challenges. These disparities necessitate a multifaceted, culturally informed approach that encompasses enhanced access to care, preventive strategies, and collaboration with tribal health organizations to mitigate the burden of TBI and improve long-term outcomes.

## Introduction

Traumatic brain injury (TBI) is a disruption in normal brain function resulting from an external mechanical force, such as a blow, jolt, or penetrating injury to the head [1]. It is characterized by a range of neurological impairments that may be temporary or permanent, affecting cognitive, behavioral, emotional, and physical functioning. The severity of TBI ranges from mild—commonly referred to as a concussion—to moderate and severe forms, which may result in prolonged unconsciousness, permanent disability, or death [2, 3]. Patients may experience a broad spectrum of acute and chronic consequences, including increased intracranial pressure (ICP), motor impairments, cognitive dysfunction, psychological and behavioral disturbances, communication challenges, sensory alterations, seizure activity, and sleep disorders, regardless of the severity of TBI [4, 5]. These sequelae contribute to long-term disabilities and negatively impact the quality of life (QoL).

Globally, about 69 million individuals sustain a TBI each year. The highest incidence rates per 100,000 population are reported in North America (1,299) and Europe (1,012), while the lowest rates are observed in Africa (801) and the Eastern Mediterranean region (897) [6]. According to the Brain Trauma Foundation, an estimated 2.5 million individuals are diagnosed with TBI annually [7]. Data from the Centers for Disease Control and Prevention (CDC) indicate that in 2020 alone, there were approximately 214,000 TBI-related hospitalizations in the United States [8]. In 2017, TBI accounted for approximately 2.2% of all mortalities in the United States and contributed to nearly 30% of all injury-related fatalities [9]. By 2021, there were 69,473 TBI-related deaths nationwide. That year, the age-adjusted mortality rate associated with TBI reached 18 per 100,000, representing a 6.5% increase from the 2019 rate of 16.9 per 100,000 [10]. These statistics highlight the substantial burden of TBI on the healthcare system and underscore the critical importance of enhancing public awareness, implementing effective prevention strategies, and ensuring appropriate clinical management of TBI patients.

Persistent racial and ethnic disparities remain a serious challenge within the U.S. healthcare system, with minority populations experiencing significantly poorer management and higher rates of hospitalizations and readmissions [11]. Evidence indicates that racial and ethnic minorities bear a disproportionate burden of TBI, with American Indian/Alaska Native (AI/AN) populations exhibiting the highest rates of TBI-related mortality and hospitalizations, with limited access to rehabilitation and post-acute care [12–19].

Globally, rural areas frequently face a shortage of trained neurosurgical personnel, compounding the challenges of managing TBI in high-burden settings [20]. In the United States, similar challenges are evident as higher rates of TBI-related morbidity and mortality have been observed in rural regions, where contributing factors are often systemic, environmental, and logistical [21]. Additionally, the recruitment and retention of healthcare providers in rural communities remain a persistent obstacle [22, 23]. Alaska — situated in the far northwestern region of North America — is a striking example of all previously mentioned challenges. Alaska is the largest and most sparsely populated state in the United States, encompassing over 663,000 square miles [8], characterized by substantial geographic and climatic diversity, including more than thirty mountain ranges and the Aleutian Islands—a 300-mile-long chain of volcanic islands. This vast and varied terrain presents significant challenges to delivering healthcare services and maintaining consistent communication across the state. The geographic, environmental, and cultural complexities of the region are critical considerations when evaluating the delivery and optimization of neurosurgical care in Alaska [24, 25]. These factors contribute to deviations from conventional neurosurgical practices commonly observed in the continental United States. As a result, significant gaps and logistical challenges persist in the provision of neurosurgical services within this predominantly rural and remote state [21].

The systematic review aimed to characterize and assess (1) Demographic patterns, including age, sex, and race/ethnicity; (2) Mechanisms of injury, such as motor vehicle accident (MVA), falls, assaults, and self-harm; (3) Hospitalization rates and clinical outcomes following TBI; (4) mortality rates and contributing causes of death, among AI/ANs in the United States, especially those residing in the state of Alaska. We also highlighted the current preventive and interventional initiatives aimed at reducing the burden of TBI among these populations.

## Methods

### Systematic literature review

Following the PRISMA 2020 (Preferred Reporting Items for Systematic Reviews and Meta-Analyses) guidelines, we conducted a systematic review of the Medline/PubMed, Google Scholar, Web of Science, Scopus, and Embase electronic databases to synthesize the available epidemiological evidence on TBI among AI/ANs in the United States [26]. A comprehensive and systematic search strategy was developed to ensure the inclusion of all relevant literature. A combination of free-text keywords was employed to capture variations in terminology related to TBI and the target population. The search focused on identifying studies involving AI/ANs in the United States and addressing epidemiology, health disparities, or interventions related to TBI. The following search terms and their variations were used: “Traumatic Brain Injury,” “TBI,” “Head Injury,” “Brain Injuries,” “Alaska,”, “US”, “USA”, “United States”, “American Indian,” “Alaska Native,” “Epidemiology,” “Healthcare Disparities,” and “Interventions.”

### Eligibility criteria

Studies were included if they met the following criteria: (1) published in peer-reviewed journals; (2) written in English; (3) focused on TBI among adults (> 18 years) AI/ANs in the United States; (4) addressed one or more of the following domains: incidence, prevalence, hospitalization, and mortality rates, mechanisms of injury, clinical outcomes, healthcare disparities, and public health or clinical interventions; (5) reported primary data or robust secondary analyses derived from recognized surveillance systems, such as CDC reports, trauma registries, or other population-based data sources. Review articles, guidelines, case reports, or studies that did not meet the inclusion criteria were excluded from the final analysis.

### Data extraction

Two authors (LS and MF) independently reviewed the literature to identify studies eligible for inclusion in the systematic review. Any discrepancies regarding study selection were resolved through adjudication by the senior author (SCP) until consensus was achieved. Each study underwent an initial eligibility assessment based on a review of its title and abstract. Articles deemed relevant to the scope of the review were subsequently subjected to full-text evaluation to assess the availability and adequacy of data per the predefined inclusion criteria.

### Data synthesis

Data synthesis involved collecting pertinent information from the selected articles, including study design, sample size, participant demographics, key findings, and any TBI-related epidemiologic data. Data from the included studies were systematically organized into a spreadsheet to facilitate subsequent analysis. A qualitative synthesis was conducted to identify recurrent themes and patterns across the literature, involving a comparative evaluation of study methodologies, participant demographics, and principal findings to construct a coherent narrative summary of the existing evidence. The findings are reported in a comprehensive narrative synthesis organized around key themes, highlighting epidemiological trends, healthcare disparities, and potential interventions.

### Quality assessment

To assess the methodological quality and risk of bias of the included studies in this systematic review, we used the NIH Quality Assessment Tool for Observational Cohort and Cross-Sectional Studies, developed by the National Heart, Lung, and Blood Institute (NHLBI) [27]. The tool includes 14 criteria that cover aspects such as clarity of the research question and study population, participation rate and inclusion criteria, reliability and validity of exposure and outcome measures, adequacy of sample size and statistical analysis, and control for confounding variables. Each study was independently rated as follows: **Good** (low risk of bias, meeting most criteria), **Fair** (some risk of bias or methodological concerns), or **Poor** (significant bias or insufficient methodological detail).

#### Rationale for not conducting a Meta-analysis

A meta-analysis was not feasible for this review due to substantial heterogeneity across the included studies in multiple key domains. There was a wide variation in study design — cross-sectional surveys, retrospective trauma registry analyses, and nationwide population-based surveillance reports — and population scope—ranging from local or state-level cohorts to national datasets. The studies employed different inclusion criteria for severity (mild, moderate, severe, or all TBIs combined). Inconsistent outcome measures: some reported age-adjusted mortality rates, others focused on hospitalization or ED visit rates, and some assessed only specific mechanisms of injury. Additionally, the time frames covered by the studies ranged from single-year snapshots to multi-decade trend analyses, and the demographic stratifications (e.g., age categories, race/ethnicity groupings, sex) were neither standardized nor consistent. This lack of alignment in data definitions and reporting metrics precluded the calculation of directly comparable effect sizes or summary estimates.

Given these methodological and definitional differences, pooling the results in a single quantitative meta-analysis would risk producing misleading summary measures. Instead, the findings are synthesized narratively to highlight consistent patterns and disparities—particularly the disproportionate TBI burden among AI/ANs—while preserving the contextual nuances of each study.

## Results

**Fig. 1:**
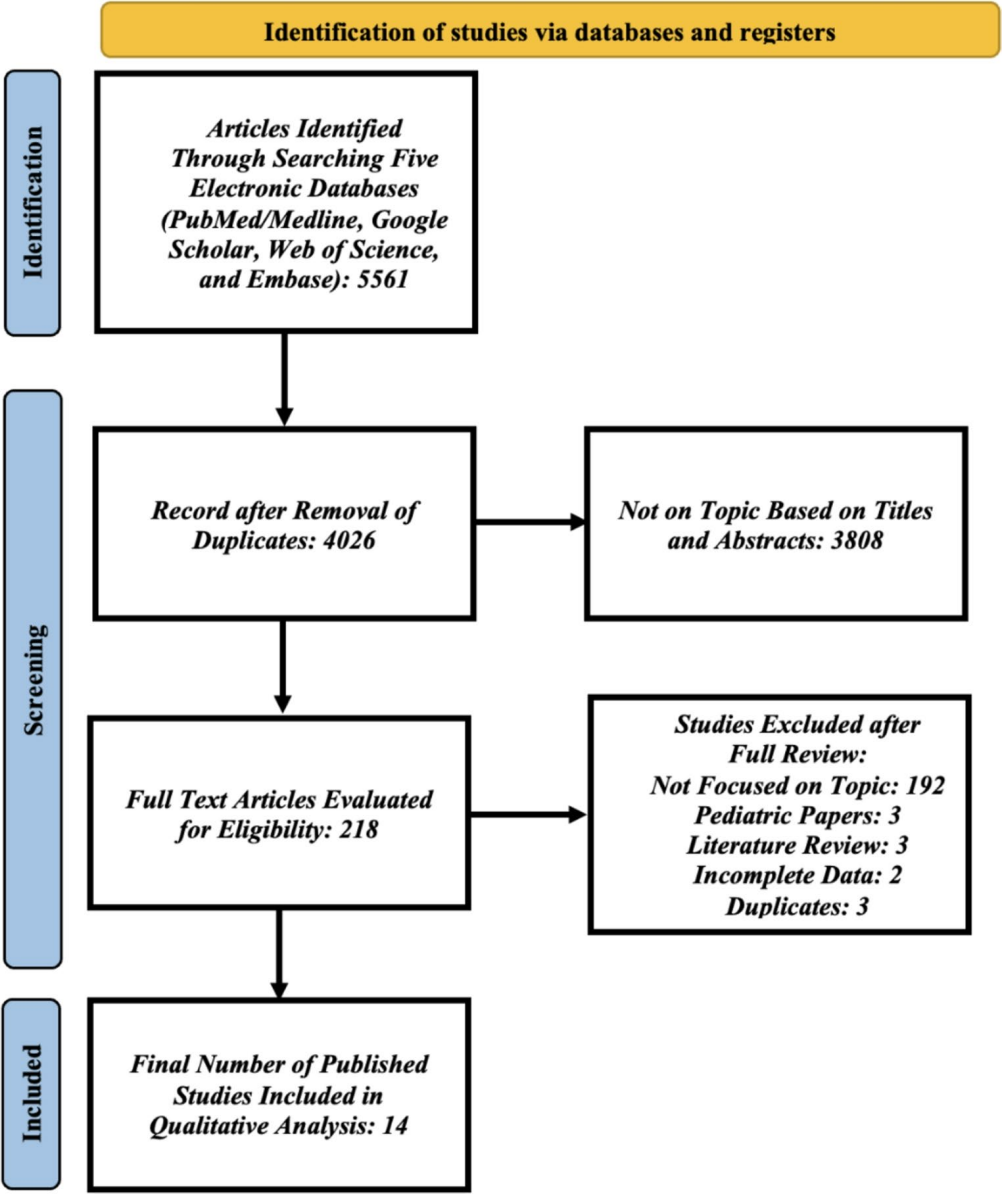
PRISMA (Preferred Reporting Items for Systematic Reviews and Meta-analysis) Flow Diagram

After a full-text review, 14 papers met the inclusion criteria and were included in the final analysis; Fig. 1. Of the included studies, three were based on CDC surveillance data [28–30], and eleven were retrospective cross-sectional studies [8–10, 12–14, 31–35]. Five studies were conducted at the state level [8, 14, 30, 33, 34], while nine were nationwide in scope [9, 10, 12, 13, 28, 29, 31, 32, 35]. Four studies explicitly reported the severity of TBI [12, 30, 31, 33]; Tables 1 and 2.

**Table 1:** Study characteristics

Study	Study Design	Data Source	Coverage
^**28**^**Adekoya et al.**,** 2002**	**CDC PBS**	CDC Surveillance	Nation Wide
^**32**^**Adekoya et al.**,** 2002**	Retrospective Cross-sectional	IHS	Nation Wide
^**30**^**Langlois et al.**,** 2003**	**CDC PBS**	CDC Surveillance	**14 States**
^**12**^**Bazarian et al.**,** 2005**	Retrospective Cross-sectional	NHAMCS	Nation Wide
^**14**^**Rutland-Brown et al.**,** 2005**	Retrospective Cross-sectional	TBI Surveillance System	**13 States**
^**31**^**Bowman et al.**,** 2007**	Retrospective Cross-sectional	NTDB	Nation Wide
^**29**^**Coronado et al.**,** 2011**	**CDC PBS**	CDC Surveillance	Nation Wide
^**34**^**Linton et al.**,** 2015**	Retrospective Cross-sectional	Arizona Trauma Database	**Arizona**
^**33**^**Hoopes et al.**,** 2015**	Retrospective Cross-sectional	Washington CHARS	**Washington**
^**9**^**Peterson et al.**,** 2019**	Retrospective Cross-sectional	HCUP NIS	Nation Wide
^**13**^**Daugherty et al.**,** 2019**	Retrospective Cross-sectional	NVSS	Nation Wide
^**35**^**Sarmiento et al.**,** 2020**	Retrospective Cross-sectional	IHS NPIRS	Nation Wide
^**10**^**Peterson et al.**,** 2022**	Retrospective Cross-sectional	NVSS	Nation Wide
^**8**^**Newell et al.**,** 2025**	Retrospective Cross-sectional	Alaska Trauma Registry	**Alaska**

^1^CDC PBS: Centers for Disease Control and Prevention, Population-based Surveillance

^2^ IHS: Indian Health System

^3^ NHAMCS: National Hospital Ambulatory Medical Care Survey

^4^ NTDB: National Trauma Data Bank

^5^ CHARS: Comprehensive Hospital Abstract Reporting System

^6^ HCUP NIS: The Healthcare Cost and Utilization Project’s National Inpatient Sample

^7^ NVSS: National Vital Statistics System

^8^ IHS NPIRS: Indian Health Service’s National Patient Information Reporting System

**Table 2:** Study key findings

Study	Key Findings
^**28**^**Adekoya et al.**,** 2002**	♣ TBI-related mortality rates were highest among AI/ANs, with 27.2 deaths per 100,000 population, followed by African Americans (25) and Whites (20.1). ♣ Among AI/ANs, firearm-related injuries represented the second leading cause of TBI-related deaths, with suicides accounting for 63% of these cases. ♣ MVA-related TBI death rates were highest among AI/ANs at 12.5 per 100,000, followed by Whites (7.2) and African Americans (6.3).
^**32**^**Adekoya et al.**,** 2002**	♣ The average annual age-adjusted TBI-related hospitalization rate among AI/ANs was 81.7 per 100,000. ♣ The leading causes of TBI among AI/ANs were MVAs (24%), assaults (17%), and falls (16%). MVA-related TBIs are most common among those aged 15–24, whereas assaults are predominant in adults aged 25–44.
^**30**^**Langlois et al.**,** 2003**	♣ The overall age-adjusted rate of TBI-related hospitalizations was 69.7 per 100,000 population. Rates were highest among AI/ANs at 75.3 per 100,000 and African Americans at 74.4 per 100,000. ♣ Assault-related TBIs were most prevalent among African American and AI/AN males, with rates of 31.3 and 29.5 per 100,000, respectively—approximately four times higher than the rate observed in White males.
^**12**^**Bazarian et al.**,** 2005**	♣ Between 1998 and 2000, the average annual incidence of mild TBI in the United States was 503.1 per 100,000 population. The highest rates were observed among AI/ANs (1,026 per 100,000), followed by African Americans (624.6), and Whites (491).
^**14**^**Rutland-Brown et al.**,** 2005**	♣ The overall age-adjusted TBI hospitalization rate among AI/ANs was 71.5 per 100,000—the highest among all racial groups. ♣ MVAs were the leading cause of TBI-related hospitalization among AI/ANs, with a rate of 27.0 per 100,000. ♣ Among AI/ANs who had MVA-related TBI, the proportion of those individuals with high blood alcohol concentrations was significantly higher than that of other racial groups.
^**31**^**Bowman et al.**,** 2007**	♣ African Americans, Hispanics, and AI/ANs were less likely to receive treatment at a level II trauma center, compared to their White counterparts.
^**29**^**Coronado et al.**,** 2011**	♣ AI/ANs exhibited the highest average annual rate of TBI-related mortality at 27.3 deaths per 100,000 population, followed by African Americans (19.3), Whites (18.8), and Hispanics (14.4). ♣ AI/ANs also had the highest average annual rate of MVA-related TBI deaths, at 11.5 per 100,000, compared to Whites (6.1), African Americans (5.4), and Hispanics (5).
^**34**^**Linton et al.**,** 2015**	♣ AI/ANs are also more than three times as likely as Whites to sustain a violent TBI and are disproportionately affected by blunt-force trauma. ♣ Compared to individuals of other racial groups, AI/ANs are more likely to present with signs of intoxication. Furthermore, their average blood alcohol concentration levels were significantly higher than those of other racial groups.
^**33**^**Hoopes et al.**,** 2015**	♣ Compared to Whites, AI/ANs were significantly younger (mean age: 36 vs. 47 years), at higher risk of severe TBI (20.7% vs. 16.8%), with a higher incidence of intentional injuries, including suicide and homicide (20.1% vs. 6.7%). They were also less likely to have used safety measures such as seat belts or airbags at the time of injury (53.9% vs. 76.7%).
^**9**^**Peterson et al.**,** 2019**	♣ Between 2008 and 2014, an estimated 14,544 traumatic brain injury (TBI)-related hospitalizations were recorded. During this period, the age-adjusted rate of TBI-related hospitalizations increased by 21%. ♣ The leading causes of TBI-related hospitalizations were falls (34.8 per 100,000 population), MVAs (20.8), and assaults (14.4). ♣ The age-adjusted TBI-related mortality rate rose by nearly 12%, from 22.7 per 100,000 in 2008 to 25.4 in 2014.
^**13**^**Daugherty et al.**,** 2019**	♣ In 2017, AI/AN individuals had the highest age-adjusted rate of TBI-related deaths among all racial and ethnic groups in the United States—28.3 per 100,000—compared to 19.4 for Whites, 16.6 for African Americans, 11.3 for Hispanics, and 8 for Asians. ♣ Suicide was the leading cause of TBI-related mortality within this group.
^**35**^**Sarmiento et al.**,** 2020**	♣ Among AI/ANs, the rates of emergency department visits for fall-related and assault-related traumatic brain injuries were approximately twice as high as those for other causes.
^**10**^**Peterson et al.**,** 2022**	♣ In 2020, AI/ANs had the highest age-adjusted TBI-related mortality rate at 29 per 100,000 population, while non-Hispanic Asian/Pacific Islander individuals had the lowest rate at 7.7 per 100,000. ♣ Suicide was the leading cause of TBI-related mortality within this group.
^**8**^**Newell et al.**,** 2025**	♣ AI/ANs individuals accounted for 31% of all TBI-related hospitalizations in Alaska, with an age-adjusted hospitalization rate of 2.4 times higher than that of Whites. ♣ TBI-related mortality among AI/AN Alaskans exceeded national rates for AI/AN individuals across all mechanisms of injury. ♣ The homicide-related TBI mortality rate in the state of Alaska was 3.8 times higher than the national rate for AI/ANs. ♣ Suicide-related TBI mortality was significantly higher in Alaska—28.9 per 100,000 compared to 6.4 nationally.

### Quality assessment

Eleven studies were assessed using the NIH Quality Assessment Tool for Observational Cohort and Cross-Sectional Studies. Six studies were rated as **good** quality, having met most of the NIH criteria for methodological rigor [8, 10, 13, 31, 33, 35]. These studies utilized nationally representative datasets, and they featured clearly defined populations, outcomes, and robust statistical methods. Five studies were rated as **fair** quality [9, 12, 14, 32, 34]. While these studies contributed valuable insights, they exhibited moderate limitations, including partial data coverage (e.g., data limited to Indian Health Service facilities), potential racial misclassification, or incomplete adjustment for key confounding variables. Of the fourteen studies included in the analysis, three were CDC surveillance reports that are not eligible for this assessment [28–30]; Table 3.

**Table 3:** NIH quality assessment tool for observational cohort and Cross-Sectional studies

Criteria	^28^ Adekoya et al., 2002	^32^ Adekoya et al., 2002	^30^ Langlois et al., 2003	^12^ Bazarian et al., 2005	^14^ Rutland-Brown et al., 2005	^31^ Bowman et al., 2007	^29^ Coronado et al., 2011	^34^Linton et al., 2015	^33^ Hoopes et al., 2015	^9^ Peterson et al., 2019	^13^ Daugherty et al., 2019	^35^ Sarmiento et al., 2020	^10^ Peterson et al., 2022	^8^ Newell et al., 2025
**Was the research question or objective clearly stated?**	N/A	Yes	N/A	Yes	Yes	Yes	N/A	Yes	Yes	Yes	Yes	Yes	Yes	Yes
**Was the study population clearly specified and defined?**	N/A	Yes	N/A	Yes	Yes	Yes	N/A	Yes	Yes	Yes	Yes	Yes	Yes	Yes
**Was the participation rate of eligible persons at least 50%?**	N/A	N/A	N/A	N/A	N/A	N/A	N/A	N/A	N/A	N/A	Yes	Yes	Yes	Yes
**Were all the subjects selected or recruited from the same or similar populations?**	N/A	Yes	N/A	Yes	Yes	Yes	N/A	Yes	Yes	Yes	Yes	Yes	Yes	Yes
**Was a sample size justification or power description provided?**	N/A	No	N/A	No	No	No	N/A	No	No	No	Yes	Yes	Yes	Yes
**For the analyses**,** were the exposure(s) of interest measured prior to the outcome(s)?**	N/A	N/A	N/A	N/A	N/A	Yes	N/A	Yes	Yes	Yes	N/A	N/A	N/A	N/A
**Was the timeframe sufficient so that one could reasonably expect to see an association?**	N/A	Yes	N/A	Yes	Yes	Yes	N/A	Yes	Yes	Yes	Yes	Yes	Yes	Yes
**For exposures of interest**,** were they clearly defined**,** valid**,** and reliably measured?**	N/A	Yes	N/A	Yes	Yes	Yes	N/A	Yes	Yes	Yes	Yes	Yes	Yes	Yes
**Was the exposure(s) assessed more than once over time?**	N/A	No	N/A	No	No	No	N/A	No	Yes	Yes	Yes	Yes	Yes	Yes
**Were the outcome measures clearly defined**,** valid**,** and reliable?**	N/A	Yes	N/A	Yes	Yes	Yes	N/A	Yes	No	No	Yes	Yes	Yes	Yes
**Were the outcome assessors blinded to the exposure status?**	N/A	N/A	N/A	N/A	N/A	N/A	N/A	N/A	Yes	Yes	Yes	Yes	Yes	Yes
**Was loss to follow-up after baseline 20% or less?**	N/A	N/A	N/A	N/A	N/A	N/A	N/A	N/A	N/A	N/A	N/A	N/A	N/A	N/A
**Were key potential confounding variables measured and adjusted statistically?**	N/A	No	N/A	No	No	Yes	N/A	Yes	Yes	Yes	Yes	Yes	Yes	Yes
**Were statistical methods well-described and appropriate?**	N/A	Yes	N/A	Yes	Yes	Yes	N/A	Yes	Yes	No	Yes	Yes	Yes	Yes
**Overall Quality Rating**	**N/A**	**Fair**	**N/A**	**Fair**	**Fair**	**Good**	**N/A**	**Fair**	**Good**	**Fair**	**Good**	**Good**	**Good**	**Good**

### Emergency department visit rates

From a pooled three-year sample (1998–2000) of 70,900 emergency department (ED) visits in the National Hospital Ambulatory Medical Care Survey (NHAMCS). The average annual incidence rate for mild TBI was 503.1 per 100,000 [12]. The highest rates were observed among AI/ANs (1,026 per 100,000), followed by African Americans (624.6), and Whites (491) [12]. Between 2005 and 2014, a total of 44,918 TBI–related ED visits were recorded across Indian Health Service (IHS) hospitals [35]. The overall age-adjusted rate of TBI-related ED visits among AI/ANs was 333.4 per 100,000, with a statistically significant increase in rates observed from 2008 to 2014 and an average annual percent change (APC) of 4.37 [35].

### Hospitalization rates

Between 1992 and 2021, five studies reported TBI-related hospitalization rates among AI/ANs ranging from 71.5 to 81.7 per 100,000 [9, 14, 30, 32]. The highest rate was observed in Alaska, reaching 186.7 per 100,000 [8].

### Mortality rates

This review spans more than three decades (1989–2021). Seven studies reported TBI-related mortality rates among AI/ANs in the United States, with rates ranging from 27.2 per 100,000 in 1998 to 29 per 100,000 in 2020, representing the highest rates among all racial and ethnic groups at all times [9, 10, 13, 28, 29, 32]. Notably, in Alaska, the TBI-related mortality rate was markedly higher, at 65 per 100,000 [8].

### Mechanism of injury

Adekoya et al. reported that MVAs, falls, and violence are the leading causes of TBI among AI/ANs [32]. In 2020, Sarmiento et al. reported that falls were the leading cause of TBI-related ED visits among AI/ANs, with violence being in second place [35]. However, falls accounted for a smaller percentage of total injuries among AI/AN individuals compared to White Patients (27.7% vs. 45.4%) [33]. On the contrary, Assault-related TBIs were twice as prevalent among AI/AN compared to other racial groups (21% vs. 10%) [14]. In comparison to White patients, AI/ANs sustained more injuries to the head and neck (42.1% vs. 37%) and intentional injuries—including those due to assault and self-harm— (20.1% vs. 6.7%) [33]. Compared to White patients, AI/AN trauma patients exhibited a significantly greater proportion of penetrating injuries (13.5% vs. 6.4%) [33].

Regarding hospitalization, in 2005, Rutland-Brown et al. found that MVAs are the leading cause of TBI-related hospitalization among AI/ANs, with a rate of 27 per 100,000, accounting for over 40% of all TBI-related hospitalizations and occurring at twice the rate of violence (13.8 per 100,000) [14]. However, in 2019, a nationwide study reported that the leading causes of TBI-related hospitalizations are falls (34.8 per 100,000 population), MVAs (20.8), and violence (14.4) [9].

The CDC reported that MVA-related TBI mortality rates were highest among AI/ANs at 11.5–12.5 per 100,000, followed by Whites (6.1–7.2), African Americans (5.4–6.3), and Hispanics (5) [28, 29]. Similarly, AI/ANs experienced the highest rate of fall-related TBI deaths (3.6 per 100,000), followed by Whites (3.2), Hispanics (2.7), and African Americans (2) [29]. Firearm-related injuries represented the second leading cause of TBI-related mortalities, with suicides accounting for 63% among AI/ANs [28]. Most recently, several studies reported that suicide and self-harm are emerging as the leading causes of TBI-related mortality among AI/ANs [8, 10, 13].

### Gender disparities

Males consistently exhibited higher age-adjusted TBI-related ED visit rates compared to females [12, 35]. Additionally, the overall age-adjusted rate of TBI-related hospitalizations in males was almost twice as high as in females [30]. This sex disparity was even more pronounced among African Americans (2.8:1) and AI/ANs (2.5:1) [10, 13, 30, 32]. In 2025, Newell et al. reported that in the state of Alaska, males accounted for 64.8% of TBI-related hospitalizations [8].

#### Age as a confounding factor

AI/ANs experience notable health disparities, including a life expectancy approximately five years shorter than that of the overall U.S. population (73 years vs. 78.5 years, respectively) [35]. The CDC reported that males aged 20–24 years had the highest rates of MVA-related TBI mortalities: 35 per 100,000 among AI/ANs, 23.4 per 100,000 among Whites, and 17.1 per 100,000 among African Americans [28]. In another report across 14 states, the CDC reported that among individuals aged 20 to 45 years, AI/ANs exhibited the highest TBI-related hospitalization rates, ranging from 92 to 105.2 per 100,000. These rates exceeded those of African Americans (65.3–85.2 per 100,000), Asians (30.1–41.2 per 100,000), and Whites (44.8–81.5 per 100,000) [30]. Assault-related TBIs were most prevalent among African American and AI/AN males, with rates of 31.3 and 29.5 per 100,000, respectively—approximately four times higher than the rate observed in White males [30]. Interestingly, in the 25–34 and 35–44-year age groups, assaults were the most common cause, with hospitalization rates of 28.2 and 23.6 per 100,000, respectively [32]. For patients younger than 15 years and those older than 45 years, falls were the leading cause of injury, with rates of 17.7 and 19.4 per 100,000, respectively [32].

#### Prevention initiatives

Most TBIs are preventable. Implementing effective prevention measures not only saves lives but also reduces long-term disability and generates significant cost savings within the healthcare system and across society at large. Implementing programs to prevent TBI, especially in high-risk activities like outdoor pursuits and sports, can reduce the incidence of head injuries. To improve healthcare services for AI/ANs, the IHS provides financial support for medical services to approximately 2.3 million AI/ANs [35]. Law enforcement strategies for the proper use of seatbelts, BAC levels less than 0.08 g/dL for drivers older than 21 years old, sobriety checkpoint programs, and awareness campaigns are effective in reducing the rates of MVAs among American Indians and Alaska Natives [35]. In 2012, the CDC launched the Stopping Elderly Accidents, Deaths, and Injuries (STEADI) initiative to provide healthcare providers with the necessary tools to identify patients at risk of falls, understand risk factors, implement effective strategies, and subsequently reduce the risk of falls among the older adult population [36].

Within the State of Alaska, TBI prevention initiatives are multifaceted, targeting a broad spectrum of safety and public health concerns. Public awareness campaigns and educational programs emphasize motor vehicle safety and the distribution of protective equipment [37–39]. Targeted interventions include fall prevention programs for older adults, such as the free provision of ice cleats and participation in balance training initiatives [40–42]. Suicide prevention strategies are similarly diverse, encompassing tribal-specific interventions, community-led initiatives, and expanded access to mental health services [43–45]. The Alaska Statewide Suicide Prevention Council partners with multiple stakeholders to address suicide rates,40 while the Alaska Native Tribal Health Consortium offers education, training, and telehealth-based support [46–48]. Injury prevention efforts also address head trauma resulting from motor vehicle collisions. The Center for Safe Alaskans (https://safealaskans.org) advances safe transportation practices through child passenger safety education, complimentary car seat inspections, and resources for novice drivers.

## Discussion

This systematic review synthesizes three decades of research on TBI among AI/ANs, drawing from both state-level and nationwide datasets. Across studies, AI/ANs consistently exhibited higher rates of TBI-related ED visits, hospitalizations, and mortality than other racial and ethnic groups. While motor vehicle accidents historically dominated as the leading cause, more recent data showed a shift toward falls, alongside persistently high rates of violence- and assault-related TBIs. Firearm-related injuries—most often associated with suicide—remain a significant contributor to mortality, and emerging evidence suggests suicide may now be the leading cause of TBI-related mortality in this population. Gender and age disparities are pronounced: males have markedly higher hospitalization and mortality rates, and younger adults—especially males aged 20–24 years—experience a tremendous burden from MVAs and assaults. These patterns are compounded by broader health inequities, including shorter life expectancy and limited access to level I trauma centers.

According to the IHS, the rate of MVA-related mortality among AI/ANs is 200% higher than that of other racial/ethnic groups [35]. In 2005, Rutland-Brown et al. reported that MVAs were the leading cause of TBI-related hospitalization among AI/ANs, accounting for over 40% of all TBI-related hospitalizations [14]. The authors also found that the proportion of AI/AN TBI patients with high blood alcohol concentrations (BAC ≥ 0.08 g/dL) was significantly higher than that of other racial groups (65.7% vs. 31.6%) [14]. In 2016, Linton et al. found that nearly half of American Indian patients (47.6%) had elevated blood alcohol levels (BAL), compared to 20.0% of White patients [49]. In contrast, 47.2% of White patients had no BAL, whereas only 14.9% of American Indian patients had no BAL [49]. Furthermore, the use of protective measures among AI/AN patients was consistently lower than that of Caucasians. Seat belt and airbag usage was reported at 53.9% among AI/ANs, compared to 76.7% among Caucasians [33]. Additionally, among AI/ANs with high BAC levels, the proportion of individuals who did not use protective equipment was substantially greater than among those with lower BAC levels for AI/ANs (93.9% vs. 58.3%) [14].

According to the National Institute of Justice, AI/AN women experience the highest rates of violence among all racial and ethnic groups in the United States [34]. AI/ANs are also more than three times as likely as Whites to sustain a violent TBI and are disproportionately affected by blunt-force trauma [50]. Compared to individuals of other racial groups, AI/ANs are more likely to present with signs of intoxication [34]. Furthermore, their average blood alcohol concentration (BAC) levels were significantly higher than those of other racial groups [34].

Several studies have shown that AI/ANs are more likely to experience a violence-related TBI compared to other racial/ethnic groups [14, 50–52]. Previous research has indicated that the prevalence of domestic violence among AI/AN populations ranges from 30 to 40%, highlighting a significant public health concern within these communities [53, 54]. The high rates of unemployment and low socioeconomic status among AI/AN communities have been reported as risk factors for suicide and intentional self-harm [55, 56]. Since 2003, the suicide rate among American Indians/Alaska Natives has been increasing, and intentional self-harm represents one of the most common causes of TBI in this community [9]. In 2015, Hoopes et al. reported that AI/ANs exhibit higher rates of intentional self-harm and interpersonal violence compared to White Americans (20,1% vs. 6.7%) [33].

We took a closer look at the state of Alaska and found that disparities in TBI-related outcomes and mechanisms of injury were significantly more pronounced in Alaska. In 2021, Daugherty et al. reported that the overall U.S. TBI-related mortality rate was 17.3 per 100,000 population per year, with the highest rate observed in Alaska (34.8 per 100,000). The national rate of intentional TBI-related deaths was 8 per 100,000, again peaking in Alaska at 17.7 per 100,000. For specific mechanisms, suicide-related TBI mortality was 7.1 per 100,000 nationally compared with 15.7 per 100,000 in Alaska, while violence-related TBI mortality was 1.4 per 100,000 nationally versus 4.3 per 100,000 in Alaska [21]. In 2025, Newell et al. found that TBI-related mortality among AI/ANs in the state of Alaska is twice the national rate for AI/ANs across all mechanisms of injury [8]. AI/ANs, who make up 15.7% of Alaska’s population, account for 31% of all TBI-related hospitalizations in the state of Alaska, with an age-adjusted hospitalization rate 2.4 times higher than that of Whites—186.7 per 100,000 compared to 73.3 per 100,000. AI/ANs experience TBI-related mortality at a rate nearly twice that of their White counterparts—65.9 vs. 33.0 per 100,000. Suicide was the leading cause of TBI-related mortality in Alaska, with AI/AN populations disproportionately affected [8]. Suicide-related TBI mortality was significantly higher than the national rate for AI/ANs—28.9 vs. 6.4 nationally [8]. Similarly, the homicide-related TBI mortality rate in the state of Alaska was 3.8 times higher than the national rate for AI/ANs. While national data consistently show AI/ANs having the highest TBI-related hospitalization and mortality rates, these disparities are even more stark in Alaska, where the racial gap in TBI mortality exceeds national averages [8]. The TBI-related death rate among AI/AN Alaskans is double that of White Alaskans, primarily due to suicide and MVAs, which together account for 64% of all TBI-related deaths among AI/ANs [8].

The effective management of TBI — and, more broadly, the delivery of standard neurosurgical care — presents substantial challenges within the state of Alaska [25]. A key limitation is the lack of access to essential diagnostic infrastructure; for instance, many hospitals do not have computed tomography (CT) scanners, despite CT imaging being a critical component of the initial assessment and diagnosis of suspected TBI cases [25]. Furthermore, geographic factors play a significant role in influencing both the incidence and outcomes of TBI [25]. Individuals residing in rural regions of Alaska experience disproportionately higher rates of TBI-related hospitalizations and mortality compared to their counterparts in urban areas, highlighting the compounded impact of limited healthcare access and environmental risk factors on this vulnerable population [24]. In regions lacking primary neurosurgical centers, patients requiring specialized care must be transferred to the nearest facility equipped with comprehensive neurosurgical services and available personnel [57–59]. Many rural hospitals in Alaska are not accessible by road and, therefore, rely on aeromedical transport for interregional patient transfers. However, weather conditions significantly contribute to unsafe flying around Alaska. According to the National Institute for Occupational Safety and Health, commercial pilots operating in Alaska experience a mortality rate five times higher than that of their counterparts in other regions of the United States [60]. Remote areas, such as Alaska and Hawaii, face substantial logistical challenges related to patient transport, particularly in the context of high-acuity TBI cases [61]. These difficulties are further exacerbated by extreme weather conditions and frequent storm events, which can significantly impede timely access to specialized medical care.

In addition to substantial transportation barriers, Alaska faces critical shortages in its neurosurgical workforce. The state has a limited number of neurosurgical providers, with only three neurosurgical services covering most of its vast territory [25]. Compounding this issue is the absence of an in-state neurosurgical residency program, which restricts opportunities for workforce development and local retention of specialized talent [62]. As of 2018, AI/ANs comprised only 0.1% of male neurosurgeons nationwide, with no reported female neurosurgeons from this racial group [63]. Beyond the shortage of neurosurgical services and the physical barriers to accessing care, there also exists a well-documented mistrust among AI/AN populations toward predominantly non-Native healthcare providers — an additional factor that further complicates efforts to deliver effective and culturally competent neurosurgical care in the region [64].

### Limitations

This systematic review is subject to several limitations stemming from the methodological and data-related constraints of the included studies. **First**, most studies relied on secondary analyses of surveillance or administrative datasets (e.g., National Vital Statistics System, National Hospital Ambulatory Medical Care Survey, Indian Health Service records, trauma registries). Such data sources are susceptible to incomplete reporting, coding errors, and misclassification of both injury characteristics and patient demographics, particularly in terms of race and ethnicity. Misclassification of AI/ANs has been well documented and can lead to systematic underestimation or overestimation of rates. **Second**, there was significant heterogeneity in case definitions for TBI across studies, including differences in International Classification of Diseases (ICD) coding versions, diagnostic inclusion criteria, and severity thresholds. This variability limits direct comparability of incidence and mortality estimates and precludes precise meta-analytic pooling. **Third**, several datasets excluded pre-hospital deaths or non-hospitalized cases, which may underrepresent fatal and mild TBIs. Conversely, studies limited to emergency department visits or hospitalizations do not capture long-term outcomes. **Finally**, important confounding factors such as socioeconomic status, comorbidities, and access to rehabilitation services were inconsistently reported or controlled for. For example, disparities in post-acute care access by race were under-investigated across almost all studies. Taken together, these limitations suggest that while the body of evidence consistently indicates a disproportionate TBI burden among AI/AN populations, the magnitude of disparities should be interpreted with caution. Future research should prioritize standardized case definitions, improved classification of race and ethnicity, and consistent reporting of key confounders to better inform prevention strategies.

### Future directions

Managing TBI in Alaska presents distinct challenges, mainly due to the state’s vast geography, remote communities, and limited healthcare infrastructure. Recovery following TBI is often compromised by a shortage of medical professionals, constrained access to specialized services, and the need for long-distance travel to reach qualified rehabilitation centers. These barriers are particularly pronounced among AI/AN populations, who face a higher risk of long-term physical and cognitive impairments due to inadequate access to timely and appropriate neurosurgical care. Addressing the complexities of TBI management in Alaska necessitates a comprehensive, multifaceted strategy that integrates technological innovations, community-based engagement, improvements in healthcare infrastructure, targeted physician recruitment, and culturally sensitive care models.

Key approaches that may contribute to improved outcomes in TBI care across the state include the following: **(1) Research and Data Collection**: Gathering comprehensive data on TBI in Alaska can provide insights into prevalent causes, risk factors, and outcomes, enabling targeted interventions; **(2) Training and Education**: Providing training to healthcare professionals in remote areas on TBI management can help them recognize and respond to head injuries effectively. Other studies have sought to address these disparities in trained providers, given the increasing demand, by suggesting that alternative non-MD personnel be trained in high-acuity and stabilizing neurosurgical procedures [65]; **(3) Community Health Workers**: Training local community health workers to provide basic education, monitoring, and support for individuals with TBI can be beneficial in areas with limited access to healthcare services: **(4) Support for Caregivers**: Providing resources and support for caregivers of TBI patients, including education on care techniques and emotional support, can improve patient outcomes; **(5) Telemedicine and Telehealth Services**: Given the remote nature of many communities, telemedicine can be a valuable tool for providing medical consultations, monitoring patients, and offering rehabilitation services remotely; **(6) Mobile Medical Units**: Deploying mobile medical units equipped with advanced medical technology can bring critical care to remote areas, ensuring that patients receive timely assessment and treatment; **(7) Emergency Medical Services (EMS) Improvements**: Enhancing emergency response capabilities and communication systems can ensure that patients receive timely care, even in remote locations; **(8) Expanded Rehabilitation Services**: Expanding rehabilitation services to remote areas through mobile clinics or telehealth can help individuals with TBI access vital therapies; **(9) Community Awareness Campaigns**: Raising awareness about TBI, their prevention, and the importance of seeking timely medical care can empower communities to take proactive measures; **(10) Culturally Sensitive Care**: Collaborating with Native and Indigenous communities to develop culturally sensitive care approaches that respect traditional practices and beliefs can improve patient engagement and outcomes; **11) Collaboration with Tribal Health Organizations**: Partnering with tribal health organizations to tailor TBI management strategies to the specific needs of Alaska Native populations; **12) Collaboration between State and Federal Agencies**: Collaborating between state and federal agencies can help allocate resources effectively to address TBI-related challenges; **13) Transportation Solutions**: Exploring innovative transportation solutions, such as air ambulance services, to swiftly transport patients in need of specialized care to medical facilities; **14) Integration of Mental Health Services**: Recognizing the psychological impact of TBI and integrating mental health services into TBI care plans is crucial for holistic recovery.

## Conclusion

American Indian and Alaska Native populations in the United States, particularly those who are residing in Alaska, are increasingly and disproportionately affected by TBI, experiencing worse outcomes and more severe long-term sequelae compared to other racial/ethnic groups. This heightened vulnerability is shaped by the state’s unique geographic and demographic characteristics, compounded by critical deficiencies in neurosurgical infrastructure, limited availability of specialized equipment, and a scarcity of neurosurgical providers. Barriers, including limited local healthcare resources, extended travel times to regional medical centers, and patient hesitancy toward non-Native physicians, further hinder efforts to optimize TBI care and implement effective preventive strategies.

To improve TBI management for AI/ANs, especially in Alaska, a multifaceted approach is essential. This includes leveraging telemedicine for remote consultations, deploying mobile medical units to serve isolated communities, and increasing public awareness about TBI prevention and the importance of timely care. Additionally, it is vital to develop culturally appropriate care models that honor Alaska Native traditions and values and to collaborate with tribal health organizations and local partners to implement targeted, community-specific interventions. Enhancing emergency response systems — particularly through expanded air ambulance services — is also crucial. Ultimately, more evidence-based, contextually tailored interventions are needed to address the leading causes of TBI and reduce its burden among AI/AN populations in Alaska.

## Data Availability

No datasets were generated or analysed during the current study.
